# Improving Laser Powder Bed Fusion IN718 Process Development Efficiency by Eliminating Pore Defects of Specified Size

**DOI:** 10.3390/ma18091929

**Published:** 2025-04-24

**Authors:** Yuzhong Wang, Wenhua Guo, Wenxian Li, Yaru Zhang, Kaiyue Ma, Qianyu Ji, Rui Han, Yihui Zhang, Chenwei Wang, Sihang Zhao, Bingheng Lu

**Affiliations:** 1 School of Mechanical Engineering, Xi’an Jiaotong University, Xi’an 710049, China; wyz_john_wang@163.com (Y.W.); jackfrost@stu.xjtu.edu.cn (W.L.); yr093333@163.com (Y.Z.); kaiyuema@stu.xjtu.edu.cn (K.M.); qianyuji@xjtu.edu.cn (Q.J.); harry56@stu.xjtu.edu.cn (R.H.); yhzhang@stu.xjtu.edu.cn (Y.Z.); iwangchenwei@163.com (C.W.); 2 National Innovation Institute of Additive Manufacturing, Xi’an 710300, China; whispilia@163.com; 3 State Key Laboratory for Manufacturing System Engineering, Xi’an 710054, China

**Keywords:** laser powder bed fusion (L-PBF), response surface methodology, pore defects elimination, mechanical properties, Inconel 718

## Abstract

The rapid identification of process windows in laser powder bed fusion (L-PBF) additive manufacturing garnered significant attention for its ability to reduce upfront engineering costs. This study focuses on accelerating the development of process windows by targeting the elimination of specific-size pore defects in L-PBF IN718. A novel relative density–porosity similarity evaluation method (DPSEM) is introduced to evaluate the reliability of porosity data derived from computed tomography (CT). Using the response surface method, the fully dense forming window (e.g., relative density ≥ 99%) was accurately located within a wide process parameter range (18–1000 J/mm^3^) in a single test. Comparative analysis with the relative density (RD) model highlighted differences in solution set distribution, positioning efficiency, microstructure, and performance within the process window. Results demonstrate that the proposed method effectively eliminates specified size defects (90 μm), achieving a maximum density of 99.5% alongside excellent mechanical properties, including an ultimate tensile strength of 1155 MPa and a yield strength of 908 MPa. In contrast, the RD model achieved a lower maximum density of 98.5%, with mechanical performance compromised by significant MC compound precipitation and keyhole pore accumulation, resulting in an ultimate tensile strength slightly exceeding 910 MPa.

## 1. Introduction

Metal additive manufacturing, particularly laser powder bed fusion (L-PBF), typically requires iterative testing to establish a formable process window, culminating in the production of fully dense samples (e.g., relative density ≥ 99%). This process entails substantial upfront engineering costs. Consequently, the development of rapid and cost-effective methods for identifying process windows has been a long-standing focus [1,2,3,4,5,6,7].

To optimize process parameters effectively, it is essential to establish a correlation model between process parameters and desired response variables (e.g., the relationship between power density and relative density). However, a comprehensive description of this correspondence is not immediately available or is still lacking. While discrete element models (e.g., powder materials) [8], heat-flow coupling models [9,10,11], thermal–mechanical models [12,13], heat–fluid–mechanical coupling models, microstructure evolution models, and process–structure–property (PSP) computational frameworks have been proposed [14,15,16]; these models face challenges related to computational complexity and incomplete quantitative descriptions. As a result, mechanism-based models are often impractical for process optimization.

Experimental optimization strategies based on statistical models have been widely adopted. These approaches involve generating datasets through experimental design and characterization, followed by constructing regression or machine learning models to approximate the manufacturing process [17,18,19,20,21,22,23,24,25]. This data-driven methodology enables the calculation of statistically optimized process parameters independent of physicochemical transformations [1,26,27,28,29]. However, regression models inherently require iterative optimization within confined parameter spaces, often resulting in forming parameters concentrated within narrow power density ranges [3,4,5,6,7,30,31,32,33]. This limited exploration restricts the utilization of the full capabilities of additive manufacturing equipment.

For more complex machine learning models, incorporating all four commonly used process parameters (laser power, scanning speed, hatch spacing, and layer thickness) significantly increases dataset size requirements, thereby raising upfront engineering costs [1,28]. Additionally, the commonly employed iterative testing method often uses relative density, measured via the Archimedean drainage method, as the optimization target [1,24]. However, components with varying pore defects can exhibit similar relative densities. Large-size defects, such as occasional "turnaround pores" or "end-of-track pores" [34], may not significantly affect relative density measurements, but their impact on mechanical properties [35,36,37], is considerably greater than that of smaller defects. Therefore, it is essential to iteratively refine the manufacturing process to eliminate hole defects that do not substantially influence density and to progressively discard forming schemes that could lead to such anomalies and pores. However, this iterative approach inevitably prolongs the testing cycle. Consequently, there is a critical need to develop more efficient optimization methods that can be applied under single-test conditions to streamline iterative testing and accelerate process development.

In this study, a response-targeted optimization strategy based on computed tomography (CT) is proposed, which differs from traditional approaches relying on relative density by focusing on the elimination of pore defects within specified size ranges. Additionally, a reliability verification method for CT-derived porosity data is introduced, and a response surface optimization model based on the verified data is established to identify the optimal process window. The effectiveness of the proposed optimization strategy is further demonstrated by comparing it with the conventional relative density-based model. This approach enables precise identification of the high-density forming window across a broad process parameter range (18–1000 J/mm^3^) through a single test, significantly accelerating the process development cycle. The remainder of this paper is organized as follows: Section 2 introduces the experimental setup and theoretical framework. Section 3 deals with the proposed CT-based porosity data reliability verification method, along with the response-targeted optimization model, and its performance evaluation. Section 4 discusses the results, and Section 5 concludes the study with a summary of the main findings.

## 2. Materials and Methods

### 2.1. Experiment Design

**Figure 1:**
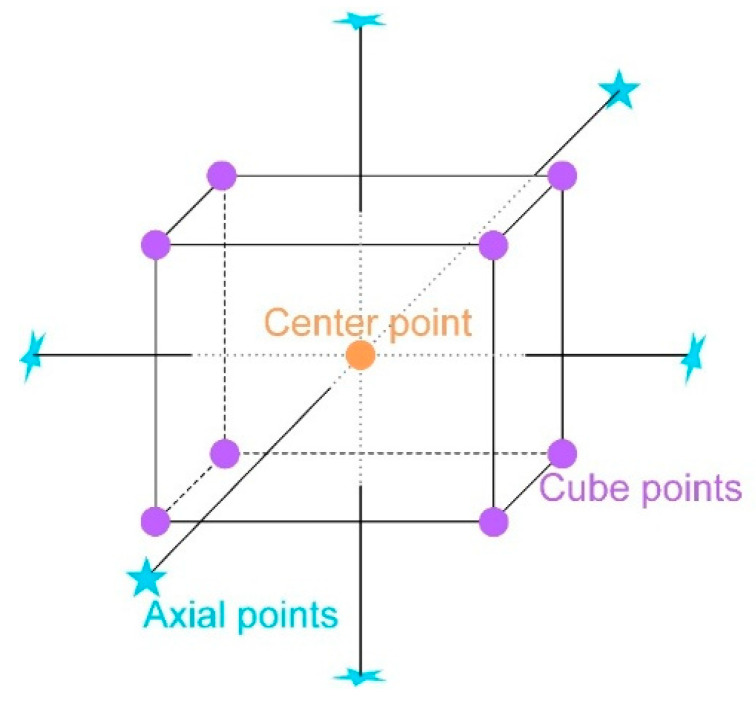
Center composite design (CCD) in three-dimensional space.

The experimental sequences involved 2 designs, including different designs with porosity and relative density (RD) as single response objectives. Different designs used the same factors: laser power (*X*_1_, W), scanning speed (*X*_2_, mm/s), hatch spacing (*X*_3_, mm), and layer thickness (*X*_4_, mm). The response surface employed the central composite design (CCD) method within a randomized design framework for all designs (Design Expert 13). CCD is widely recognized as the most extensively employed design method in response surface research. Assuming there are *k* input factors, expressed in the encoding form as *x* = (*x*_1_, *x*_2_, …, *x*_*k*_), a CCD consists of three main components: *n*_*s*_ cube points, *n*_*c*_ center points, and 2*k* axial points, as shown in Figure 1. Among these components, typically fewer than 6 center points are used and the value of the axis point usually ranges from 1 to $\sqrt{1-k}$ [38,39]. The power density (*P_d_*) resulting from the combination of all factors in the experiment ranges from 18 J/mm^3^ to 1000 J/mm^3^, as shown in Table 1; *P*_*d*_ is computed according to Equation (1).

(1) $$P_{d}=\frac{P}{vlt}$$

where *P* is the power density (W), *v* is the scanning speed (SS, mm/s), *l* is the hatch spacing (HS, mm), and *t* is the layer thickness (LT, mm).

### 2.2. Fabrication and Characterization

#### 2.2.1. IN718 Alloy Fabrication

**Figure 2:**
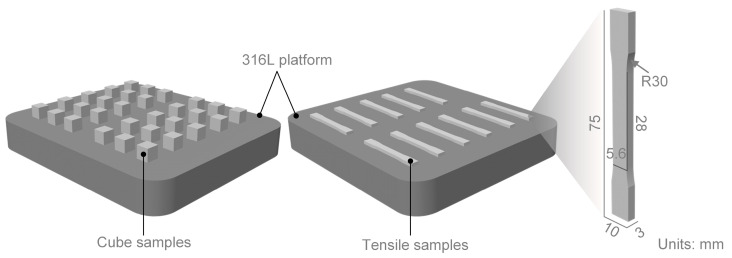
Sample forming scheme and its geometry dimensions.

The self-developed L-PBF equipment was sourced from the China National Institute of Additive Manufacturing to produce IN718 samples. The chemical composition of the powder materials is listed in Table 2. The cube samples (8 mm × 8 mm × 8 mm) and the tensile samples are manufactured parallel to the 316L steel platform. The 43 cube samples are manufactured for relative density measurement, X-ray computed tomography (CT) detection, and process window positioning verification, as shown in Figure 2. Throughout the entire process, the laser spot size was consistently maintained at 100 μm, and the inter-layer rotation was set at 67°.

#### 2.2.2. Surface Topography and Porosity Characterization

Morphological changes induced by different parameter combinations were quantitatively assessed using the versatile ZEISS Smartproof 5 widefield confocal microscope. In high-precision mode, approximately 980 slices were obtained for each sample, and the roughness curves of the surface topography were calculated.

Density measurements were conducted using the DahoMeter DH-220MN (DAHONGMEITUO measuring instrument CO., LTS., Shenzhen, China.). The theoretical density used to calculate the L-PBF IN718 relative density (RD) was 8.24 g/cm^3^.

The GE Phoenix M300 X-ray computed tomography system was used to identify pore defects. To minimize inspection costs, the focus was on eliminating defects larger than 90 *μ*m. In fact, the defect size targeted in this study is significantly smaller than the minimum acceptable defect size for L-PBF IN718, as specified in ASTM 123 WK75329 [40]. Therefore, scaling up the test target size could further reduce inspection costs while maintaining adherence to the standard. An 18 μm/voxel resolution was selected, with a source-to-detector distance of 800 mm and a 0.5 mm Cu filter. For data analysis, the EasyPore VGDefX threshold-only algorithm in VG Studio Max was employed, considering only defects exceeding 5 voxels to ensure data accuracy and reliability.

#### 2.2.3. Microstructure Characterization

Cube samples for verification were ground using a series of water-cooled SiC papers of varying grit sizes (80–2000), followed by polishing on an AutoMet 250 Grind-Polisher with low-nap cloths. Electrolytic etching was then performed with a 15% saturated solution of oxalic acid at 5 V for 3–5 s. Microstructures were observed using a Zeiss Axio Vert.A1 MET Brightfield/Darkfield Metallurgical Microscope and a JSM-7900F (JEOL Ltd., Tokyo, Japan) Schottky Field Emission Scanning Electron Microscope (SEM). For further analysis using electron backscatter diffraction (EBSD), the etched layer underwent a 40 min polishing and milling process with a triple ion beam (Leica EM TIC 3X Ion Beam Milling System, Leica Microsystems GmbH, Wetzlar, Germany). The milling vacuum level was 3 × 10^−3^ Pa for 8 h. Grain size and orientation were evaluated using Aztec Crystal software with EBSD data collected at an operating voltage of 20 kV.

#### 2.2.4. Mechanics Performance Testing

The dog bone specimen was detached from the substrate using wire cut electrical discharge machining (WEDM) and machined into the final geometry as shown in Figure 2 with surface roughness manually reduced below 3.2 μm. A uniaxial tensile test was performed on an Instron 5982 testing machine at a strain rate of 0.001 s^−1^ under ambient temperature conditions to evaluate its mechanical properties (based on Chinese national standard GB/T228.1-2021 [41]).

## 3. Results

### 3.1. Reliability Verification of Porosity

**Figure 3:**
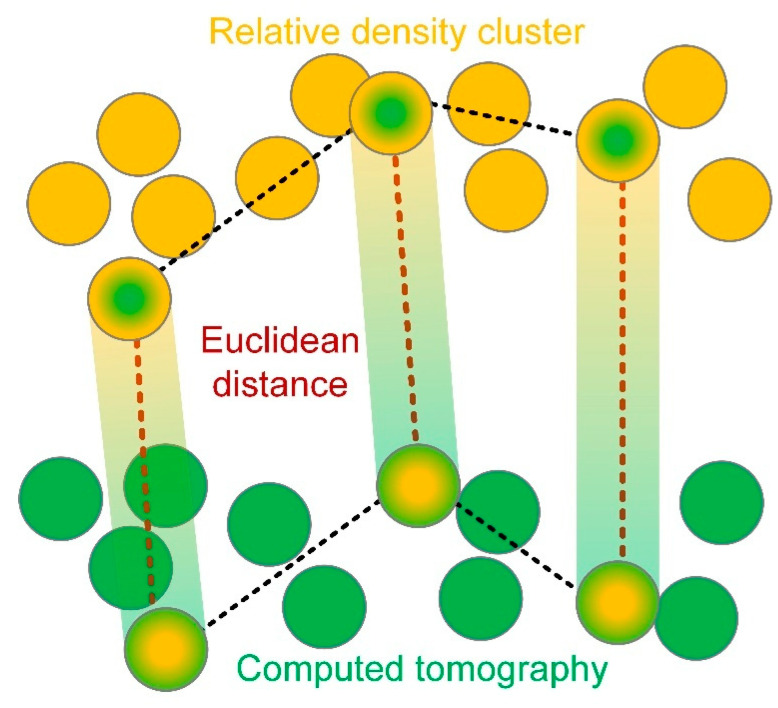
Relative density-porosity similarity evaluation method.

During CT characterization of pore defects, the flexibility of the defect labeling method based on threshold segmentation can lead to variability in data reliability, which heavily relies on the data analyst’s judgment in selecting an appropriate threshold. To mitigate this variability, particularly when porosity serves as a constraint of the statistical model, it is crucial to evaluate the measurement process. Recognizing the relationship between relative density (RD) and porosity, this study introduces a density–porosity similarity evaluation method (DDSEM) to validate CT-based porosity data. This approach ensures that the reliability of process window identification is not compromised by abnormal porosity data distributions during statistical analysis, as illustrated in Figure 3.

Following these considerations, a minimum of three randomly selected samples from the analyzed set should be tested for RD using the Archimedean drainage method, with the procedure repeated at least twice. The porosity data are then normalized, and the Euclidean distance between the RD and the porosity of each sample is calculated. Obtain at least two sets of Euclidean distance indicators and determine their cosine similarity. If the similarity between randomly selected porosity data and relative density measurements exceeds 0.98, it indicates that human error in CT image processing has a negligible impact on the intrinsic distribution characteristics of the data. Conversely, if the similarity falls below 0.98, it suggests potential inaccuracies in CT detection or data processing, necessitating a re-evaluation or repetition of these steps to ensure data reliability.

In this study, data reliability was verified by randomly selecting three sets of porosity and relative density data. All selected datasets demonstrated a cosine similarity exceeding 0.98, confirming the robustness of the CT image processing and the consistency of the data distribution characteristics, as shown in Table 3.

### 3.2. Response Surface Model

Table 4 illustrates the experimental sequence of each factor and their corresponding porosity and relative density values. Table 5 presents the ANOVA results of the experiment. The combination of the variables represents the interaction between the factors. In ANOVA, factors with *p*-values below 0.05 are generally considered statistically significant in influencing the system’s response.

**Figure 4:**
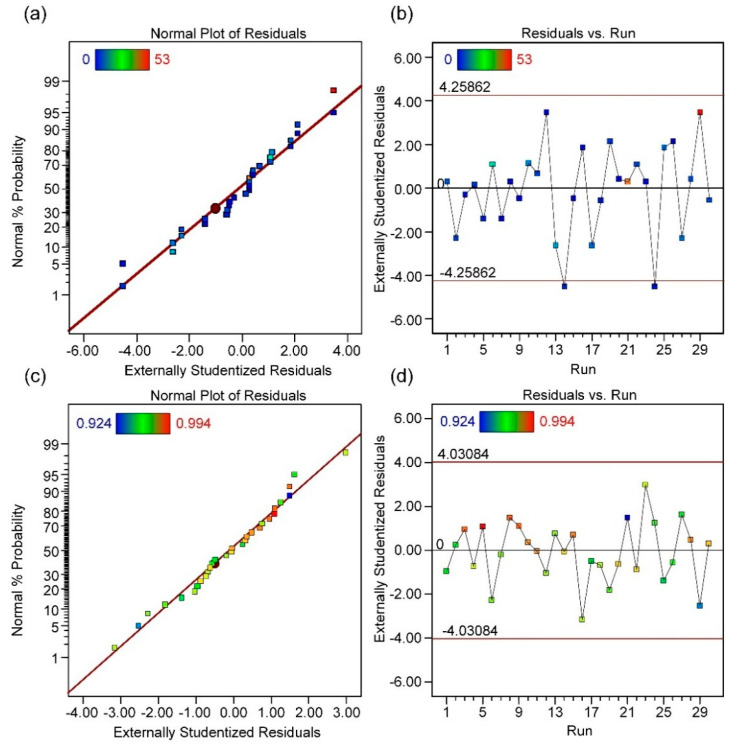
The normality test of the distribution of external studentized residuals and the test for examining the relationship between running order and residual distribution; (**a**,**b**) present the test results for the porosity model; and (**c**,**d**) present the test results for the RD model.

**Figure 5:**
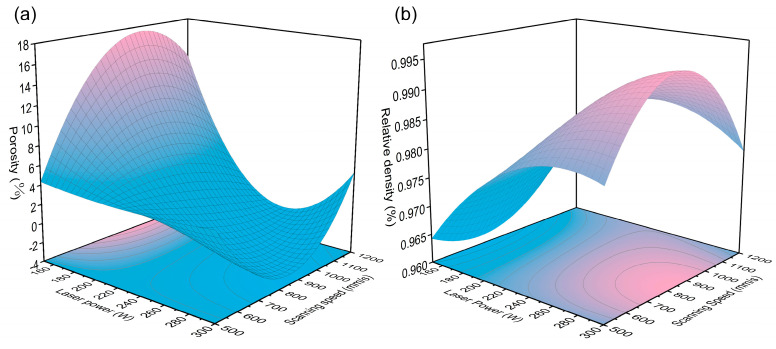
Response surface of porosity (**a**) and relative density (**b**).

The high F-values and low *p*-values for the RD and porosity models confirm the validity of the fitted models. To simplify the model, variables with negligible influence were intentionally excluded. Furthermore, an examination of the relationship between test running order and external studentized residuals reveals random distribution characteristics, suggesting that time-related variables do not threaten the objective system and that the test processes are independent, as shown in Figure 4. Additionally, the high R^2^ and adjusted R^2^ (Adj. R^2^) values further validate the reliability of both models. Based on the ANOVA results, the quantified relationships between porosity (*Y*_*p*_) and relative density (*Y*_*rd*_) are expressed in Equations (2) and (3), and the response surface corresponding to the model fitting results is shown in Figure 5.

(2) $$Y_{p}=-11.4X_{1}-1.1X_{2}+0.8X_{3}+2X_{4}-5.3X_{1}X_{2}-2.2X_{1}X_{3}-3.1X_{1}X_{4}+2.8X_{2}X_{3}+1.8X_{2}X_{4}+2.7X_{3}X_{4}+\;\;\;5.1X_{1}^{2}-0.02X_{2}^{2}-1.9X_{1}X_{2}X_{3}-2.2X_{1}X_{2}X_{4}-4.4X_{1}X_{3}X_{4}+4.6X_{2}X_{3}X_{4}+4.6X_{1}^{2}X_{2}+7.6X_{1}X_{2}^{2}+2.2$$

(3) $$\begin{array}{c} Y_{rd}=0.02X_{1}-0.001X_{2}-0.001X_{3}-0.001X_{4}+\\ \;\;\;\;0.004X_{1}X_{2}-0.002X_{1}X_{3}+0.002X_{1}X_{4}-0.004X_{2}X_{3}-0.004X_{2}X_{4}+0.003X_{3}X_{4}-0.007X_{1}^{2}+0.004X_{2}^{2}+0.004X_{1}X_{2}X_{3}+0.004X_{1}X_{2}X_{4}-0.004X_{2}X_{3}X_{4}-\\ \;\;\;\;0.012X_{1}X_{2}^{2}+1 \end{array}$$

### 3.3. Optimal Solution Set

**Figure 6:**
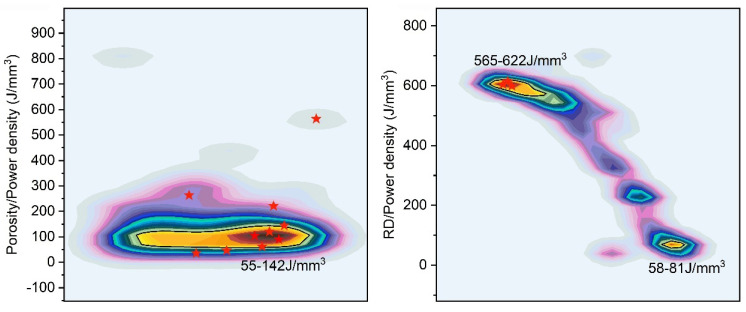
Solutions’ power density clustering of the porosity model and RD model.

The distribution of the optimal solution sets for the regression models based on porosity and relative density is presented in Figure 6. Solutions with a distribution density exceeding 60% are defined as highly reliable. The optimal solutions of the porosity model are concentrated within a distinct range of 55–142 J/mm^3^, forming an independent cluster. In contrast, the RD model solution set exhibits two distinct power density clusters: 565–622 J/mm^3^ and 58–81 J/mm^3^.

**Figure 7:**
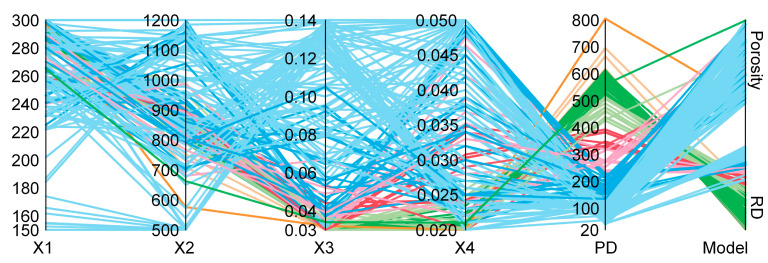
Distribution of each variable in the optimal solution set of different response models.

Unlike the solution set of the RD model, the porosity model’s process parameter combinations are dispersed across the preset intervals of individual variables. In contrast, the RD model solution set is concentrated at high power, low scanning speed, and reduced layer thickness, leading to a sharp increase in power density, as shown in Figure 7. These high-power density configurations may cause significant keyhole pore defects due to deviations from standard conduction-mode processes [42,43].

### 3.4. Formable Window Validation

To validate the reliability of the solution set, 10 representative process parameter combinations were selected from various positions within the porosity model’s solution set, as indicated by the red stars in Figure 6. Among these, P3–P7 represent samples from the high aggregation density region of the solution set. Additionally, three representative process parameter combinations were chosen from the positions with the highest aggregation density in the RD model’s solution set for further testing. The specific process parameter combinations are detailed in Table 6.

**Figure 8:**
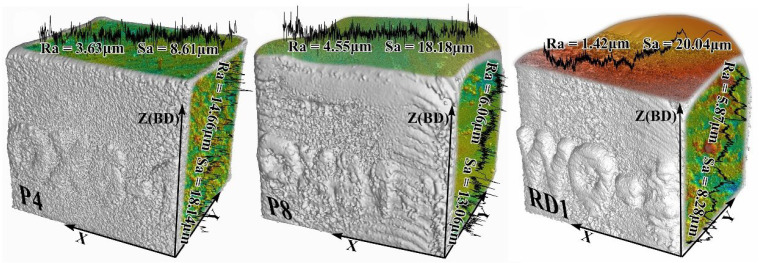
Macroscopic morphology and surface roughness of representative samples.

To intuitively illustrate the differences in the macroscopic morphology of the obtained samples, the samples’ surface characteristics were analyzed using CT and confocal microscopy to observe the macro morphology and surface roughness of representative samples (where R_a_ denotes the average line roughness on the test plane, and S_a_ denotes the average roughness of the entire tested surface), as depicted in Figure 8.

The sample optimized for porosity as the response variable (P4) accurately reproduced the designed cube geometry, with surface roughness values of R_a_ = 3.63 μm and S_a_ = 8.61 μm on the top surface, and R_a_ = 14.66 μm and S_a_ = 18.14 μm on the side surface. However, when the process parameters deviated from the solution aggregation region (P8), the sample exhibited thermal expansion, leading to increased roughness on the top surface (R_a_ = 4.55 μm, S_a_ = 18.18 μm) and reduced roughness on the side surface (R_a_ = 6.06 μm, S_a_ = 13.06 μm).

In contrast, the representative sample of the RD model experienced significant thermal expansion (Figure 8, RD1), causing a pronounced deviation from the designed cube geometry. This was accompanied by considerable fluctuations in surface roughness, with values of R_a_ = 1.42 μm and S_a_ = 20.04 μm on the top surface, and R_a_ = 5.87 μm and S_a_ = 8.28 μm on the side surface.

Furthermore, regarding the structural integrity of the formed samples, the optimization method aimed at eliminating defects of specified size did not detect any defects larger than 90 μm in the obtained samples, as detailed in Table 7. Additionally, the density measured using the drainage method (with a mean relative density of 99.4%) was notably higher than that achieved by the model optimizing for relative density (which had a mean relative density of 98.4%, and the porosity level determined by CT ranged between 0.15% and 0.28%). The regression model designed to eliminate specific-sized defects demonstrated stable performance, achieving a maximum relative density of 99.5% (P5) in a single trial. However, process parameter combinations outside the high-density region (60% concentration density) led to a decline in density (e.g., P1, P2, P8, P9, and P10), underscoring the effectiveness of the model in accurately positioning the optimized process window.

### 3.5. Microstructure

**Figure 9:**
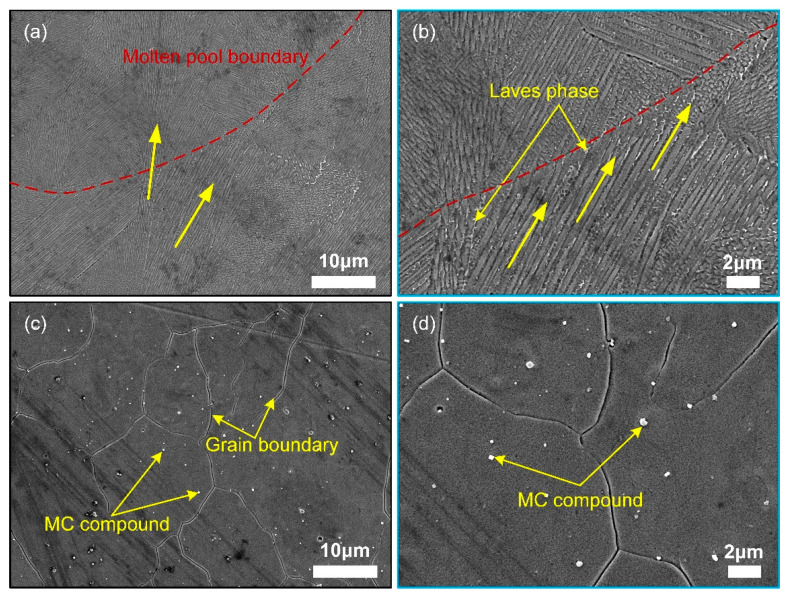
SEM characterization of the microstructure of P6 (**a**,**b**) and RD1 (**c**,**d**).

The microstructure of representative samples, positioned within a process window designed to eliminate defects of specified sizes, is illustrated in Figure 9a and highlights the typical epitaxial growth behavior of grains. Furthermore, the competitive growth behavior of typical cellular dendrites is observed, as shown in Figure 9b. Additionally, the Laves phase displays a characteristic discrete distribution along the cellular dendrites. However, no melt pool boundary was detected in the matrix of the representative sample used in the model with relative density as the response target, as illustrated in Figure 9c. Instead, significant precipitation of dispersed MC compounds was observed, as shown in Figure 9d.

**Figure 10:**
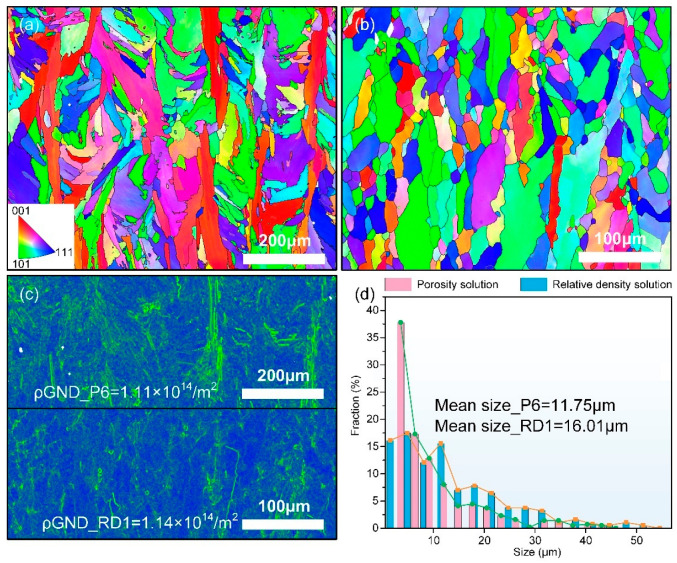
Grain orientation and kernel average misorientation (KAM) maps: (**a**) inverse pole figure map(IPF) map of sample P6; (**b**) IPF map of sample RD1; (**c**) KAM maps for samples P3 and RD1, and (**d**) grain size for samples P6 and RD1.

To compare the optimization outcomes and derive the matrix grain characteristics, the microstructure of selected representative samples was analyzed using EBSD, with the findings illustrated in Figure 10. The grain morphology of the sample derived from the computed tomography porosity model exhibits a strong correlation with the melt pool structure, displaying a typical characteristic <001> preferential growth (Figure 10a). Notably, the proportion of fine grains is markedly higher compared to the representative sample of the relative density (RD) model solution set, with an average grain size of only 11.75 *μ*m, as shown in Figure 10d. In contrast, the RD model solution set sample shows grain growth independent of the melt pool morphology, with grains elongated along the build direction and regular grain boundaries (Figure 10b), but a larger average grain size of 16.01 μm. Both samples exhibit similar geometric necessary dislocation densities (GND) ($\rho_{P6}^{GND}=1.11\times 10^{14}/m^{2}$, $\rho_{RD1}^{GND}=1.14\times 10^{14}/m^{2}$). Nevertheless, the kernel average misorientation (KAM) of the representative sample of the RD model solution set tends to concentrate at high-angle grain boundaries, as illustrated in Figure 10c.

## 4. Discussion

Although similar hyperbolic systems can be derived using both relative density and CT-based porosity, as illustrated in Figure 5, the optimal solution sets for these methods exhibit markedly different power densities and compression efficiencies for the process window. The porosity model demonstrates a preference for high-efficiency aggregation at low power densities, compressing the power density range from 18–1000 J/mm^3^ to 55–142 J/mm^3^, as shown in Figure 6. In contrast, the solution set of the RD model is distributed across two extreme communities, with a predominance of high-power density solutions. Consequently, in terms of process window positioning efficiency, the porosity model outperforms by enabling more efficient process window identification through a single test within the huge power density space (18–1000 J/mm^3^).

Furthermore, the representative sample of the model with porosity as the response target demonstrated an excellent relative density, as shown in Table 7 (P3–P7), reaching up to 99.5%, and successfully eliminated all hole defects larger than 90 μm, which were responsible for insufficient density, ensuring stable performance. However, samples deviating from the defined high-density aggregation area (P1, P2, P8, P9, and P10) exhibited noticeable density deterioration (P1, P2, P8, P9, and P10). In contrast, the relative density response model exhibits a weaker regulatory effect on scanning speed and hatching space, causing these two variables to cluster in a narrower range, resulting in a significantly higher power density of the solutions compared to the solution set of the porosity model (Figure 7). This is the primary cause of the pronounced thermal expansion observed in the representative sample of the RD model’s solution set (Figure 8), leading to keyhole mode formation and subsequent relative density insufficiency [10,44,45,46,47].

**Figure 11:**
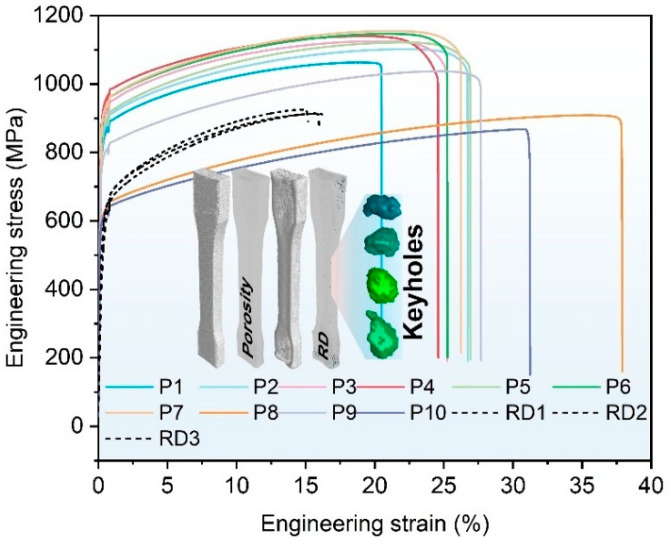
Stress–strain curves of representative samples from the porosity model and RD model.

The mechanical properties of the representative samples used to validate the reliability of the solution sets are summarized in Table 8. Samples optimized through the porosity model exhibit a dense matrix and a high proportion of fine grains (Figure 10d), resulting in excellent mechanical properties, with ultimate tensile strength reaching 1155 MPa, yield strength up to 908 MPa, and elongation of 30%. However, samples deviating from the high-density solution aggregation zone (e.g., P8 and P10) display fine interlayer grains due to excessive heat input [48,49], which increases elongation to 38%. In contrast, the solution set from the RD model suffers from significant keyhole pore defects caused by high heat input (Figure 11). These defects considerably reduce the effective load-bearing area of the samples, leading to premature fractures and a marked reduction in elongation, averaging only 16%, as illustrated in Table 8. Furthermore, the substantial precipitation of brittle MC compounds, as shown in Figure 9, exacerbates the degradation of mechanical properties [50,51], with maximum tensile strength limited to 925 MPa.

## 5. Conclusions

In this study, we propose a method to efficiently locate a formable window for achieving high relative density by eliminating pore defects of a specified size (90 *μ*m). The reliability of porosity data obtained through a threshold segmentation algorithm is validated using a proposed DPSEM. Comparative analyses were conducted using a porosity regression model and a relative density optimization response surface model. The superiority of the proposed optimization strategy was verified by solution set distribution analysis, relative density (porosity) measurement, SEM, EBSD, and property characterization. The main findings are as follows: 

The proposed process window positioning method based on eliminating the specified size defects has excellent performance for the compression efficiency of the solution set, compressing the power density range from 18–1000 J/mm^3^ to 55–142 J/mm^3^ through a single experiment. Pore defects larger than 90 μm were completely eliminated, achieving a maximum relative density of 99.5% and an average of 99.4% with stable performance. Samples optimized through the porosity model exhibited superior mechanical properties, with an average UTS of 1137 MPa and elongation of 27%. The solution set of the RD model tended to favor high power densities, resulting in keyhole mode formation with high heat input. This caused significant deterioration in elongation, averaging only 16%. Furthermore, while this model allowed for grain morphology independent of the fusion line, it also led to grain coarsening and substantial precipitation of brittle MC compounds, which adversely affected mechanical performance.

These findings highlight the superiority of the proposed optimization strategy in achieving high relative density and improved mechanical properties while maintaining process efficiency.

## Figures and Tables

**Table 1:** Value ranges of each factor level in the CCD design.

Name	Goal	Minimum	Maximum
X_1_ (Laser power, W)	Range	150	300
X_2_ (Scanning speed, mm/s)	Range	500	1200
X_3_ (Hatch spacing, mm)	Range	0.03	0.14
X_4_ (Layer thickness, mm)	Range	0.02	0.05

**Table 2:** Chemical composition of the superalloy IN718 in wt.% provided by the vendor.

Ni	Cr	Nb	Mo	Ti	Al	C	Mn	Si	Fe
53	20	5.3	3	1.05	2.5	0.03	<0.35	<0.35	Bal.

**Table 3:** Reliability verification of porosity data based on CT for 30 cubic samples involved in running CCD tests: NVP represents the normalized value of the porosity, POV represents the original data of the porosity, RDOV represents the original value of RD, and ED represents the Euclidean distance.

ED (E1)	1.6203		ED (E2)	1.6083		ED (E3)	1.4012	
NVP	POV	RDOV	NVP	POV	RDOV	NVP	POV	RDOV
0.0264	1.3900	0.9735	0.0089	0.4700	0.9742	0.3166	16.6500	0.9706
0.0352	1.8500	0.9945	0.1154	6.0700	0.9579	0.1322	6.9500	0.9658
0.0662	3.4800	0.9651	0.0004	0.0200	0.9726	0.0662	3.4800	0.9831
**Cosine similarity**			**Cosine similarity**			**Cosine similarity**		
E1 and E2		1.0000	E2 and E3		0.9974	E3 and E1		0.9976

**Table 4:** The coding level of four controllable factors and the true value of two response variables.

Run	X_1_	X_2_	X_3_	X_4_	*P_d_*	Porosity	Relative Density
1	225	150	0.085	0.035	504	4.73	0.964
2	150	1200	0.14	0.02	45	3.48	0.965
3	225	850	0.085	0.035	89	0.84	0.987
4	225	850	0.085	0.035	89	2.97	0.976
5	150	500	0.14	0.05	43	1.85	0.995
6	150	1200	0.03	0.02	208	16.65	0.971
7	300	1200	0.14	0.05	36	0.33	0.971
8	375	850	0.085	0.035	148	0.34	0.987
9	225	850	0.085	0.035	89	0.10	0.988
10	225	850	0.085	0.035	89	7.27	0.983
11	225	850	0.025	0.035	303	3.48	0.983
12	300	500	0.14	0.05	86	0.02	0.973
13	300	500	0.03	0.05	400	9.79	0.971
14	300	1200	0.03	0.02	417	2.20	0.978
15	225	850	0.085	0.035	89	0.08	0.985
16	300	1200	0.03	0.05	167	0.60	0.973
17	150	1200	0.03	0.05	83	6.07	0.958
18	225	850	0.195	0.035	39	1.39	0.974
19	300	1200	0.14	0.02	89	2.92	0.969
20	225	850	0.085	0.005	623	0.00	0.979
21	75	850	0.085	0.035	30	45.86	0.924
22	300	500	0.03	0.02	1000	3.93	0.979
23	225	1550	0.085	0.035	49	0.47	0.974
24	150	500	0.03	0.02	500	0.87	0.968
25	150	500	0.03	0.05	200	5.49	0.955
26	150	500	0.14	0.02	107	0.01	0.966
27	300	500	0.14	0.02	214	6.95	0.966
28	225	850	0.085	0.035	89	4.20	0.984
29	150	1200	0.14	0.05	18	52.59	0.935
30	225	850	0.085	0.065	48	3.91	0.980

**Table 5:** The ANOVA results of the CCD design.

Models					
RD			Porosity		
Factors	F value	*p* value	Factors	F value	*p* value
Model	7.1	0.0005	Model	10.67	0.0001
X_1_	39.49	<0.0001	X_1_	48.48	<0.0001
X_2_	0.86	0.3717	X_2_	0.42	0.5281
X_3_	0.93	0.3528	X_3_	0.66	0.4345
X_4_	0.72	0.4115	X_4_	4.41	0.0597
X_1_X_2_	4.39	0.0563	X_1_X_2_	21.23	0.0008
X_1_X_3_	1.15	0.3026	X_1_X_3_	3.61	0.0838
X_1_X_4_	0.7	0.4171	X_1_X_4_	7.38	0.02
X_2_X_3_	5.27	0.0389	X_2_X_3_	5.93	0.033
X_2_X_4_	4.76	0.0481	X_2_X_4_	2.45	0.1458
X_3_X_4_	2.52	0.1366	X_3_X_4_	5.44	0.0397
X_2_	27.57	0.0002	X_1_^2^	34.66	0.0001
X_2_^2^	7.74	0.0156	X_2_^2^	0.0006	0.9802
X_1_X_2_X_3_	5.49	0.0357	X_1_X_2_X_3_	2.75	0.1256
X_1_X_2_X_4_	3.85	0.0714	X_1_X_2_X_4_^2^	3.62	0.0835
X_2_X_3_X_4_	5.33	0.038	X_1_X_3_X_4_	14.61	0.0028
X_1_X_2_2	14.07	0.0024	X_2_X_3_X_4_	16.13	0.002
-	-	-	X_1_^2^X_2_	5.19	0.0437
-	-	-	X_1_X_2_^2^	14.46	0.0029
Lack of Fit	3.48	9.28E-02	Lack of Fit	4.04	7.35E-02
R^2^	0.9	-	R^2^	0.95	-
Adj. R^2^	0.77	-	Adj. R^2^	0.86	-

**Table 6:** Representative combination of process parameters for formable window validation.

ID	P (W)	SS (mm/s)	HS (mm)	LT (mm)	*P_d_* (J/mm^3^)
P1	298	1145	0.14	0.05	39
P2	292	1150	0.12	0.05	44
P3	184	1185	0.14	0.02	55
P4	238	1136	0.12	0.02	74
P5	230	1173	0.07	0.03	85
P6	298	924	0.10	0.02	128
P7	245	921	0.09	0.02	135
P8	282	836	0.03	0.05	220
P9	294	674	0.05	0.03	260
P10	278	674	0.03	0.02	568
RD1	300	812	0.03	0.02	616
RD2	300	815	0.03	0.02	613
RD3	300	809	0.03	0.02	618

**Table 7:** Relative density, porosity of representative samples for formable window validation.

ID	RD/%	Porosity/%
P1	99.1	4.17
P2	99.3	2.52
P3	99.4	0.00
P4	99.4	0.00
P5	99.5	0.00
P6	99.4	0.00
P7	99.4	0.00
P8	98.8	0.54
P9	99.1	0.31
P10	99.3	1.22
RD1	98.4	0.22
RD2	98.2	0.15
RD3	98.5	0.28

**Table 8:** Ultimate tensile strength (UTS), yield strength (YS), and elongation of representative samples for formable window validation.

ID	UTS/MPa	YS/MPa	Elongation/%
P1	1063	836	20
P2	1102	850	28
P3	1125	896	25
P4	1140	926	28
P5	1121	872	28
P6	1146	913	26
P7	1155	908	30
P8	909	611	38
P9	1037	784	30
P10	868	606	31
RD1	913	621	16
RD2	914	587	16
RD3	925	616	15

## Data Availability

The data presented in this study are available on request from the corresponding author. The data are not publicly available due to privacy reasons.
